# Development of a pharmaceutical care program in progressive stages in geriatric institutions

**DOI:** 10.1186/s12877-018-1002-1

**Published:** 2018-12-19

**Authors:** Conxita Mestres, Marta Hernandez, Anna Agustí, Laura Puerta, Blanca Llagostera, Patricia Amorós

**Affiliations:** 1 School of Health Sciences Blanquerna, University Ramon Llull, Padilla 326, 08025 Barcelona, Spain; 2 Pharmacy Department, Grup Mutuam, Barcelona, Spain; 3 Pharmacy Department, HSS Mutuam Girona, Girona, Spain; 4 Pharmacy Department, HSS Mutuam Güell, Barcelona, Spain; 5 Pharmacy Department EARs Grup Mutuam, Barcelona, Spain; 6 Pharmacy Department, Centre Residencial La Creueta, Sabadell, Spain

**Keywords:** Elderly, Inappropriate drugs, Drug related problems, Pharmaceutical care

## Abstract

**Background:**

To introduce and manage a Pharmaceutical care programs in geriatric care institutions presents difficulties such as reduced pharmacy service staff, complexity of the patients or lack of integration of the pharmacist in the health care team. This work describes the evolution of the implementations of a program of pharmaceutical care centered in drug related problems (DRP) in a group of geriatric institutions of different levels of complexity.

**Methods:**

Setting: Long-term and subacute care hospitals (HSS) and Health care teams attending nursing homes (EARs).

Participants: Patients attended in HSS and EARs during different periods between 2010 and 2016.

Interventions: The program was developed in different stages, in which pharmacists made interventions of increasing complexity.

**Results:**

Between 2010 and 2013, the approach was only to improve the prescription of non-appropriate drugs for the elderly, which was reduced from 19 to 14.5%. Subsequent steps included detection of drug-related problems (DRP), systematization of treatment revisions, recording of pharmacist interventions, improvements in the classification of interventions and the creation of a web-based database for recording in a more efficient way.

During these years, there was an increase in the number of patients included in pharmaceutical care activities and thus the number of pharmacist interventions (3872 in 2014 vs 5903 in 2016). In 2016, mean age in 2016: 83.2 years old. Mean number of medicines/patient: 8.4 ± 3.3, and mean interventions/patient: 1.62. Degree of acceptance of the interventions by physicians improved (68.6% in 2016 vs 45.5% in 2012), even though there is still much work to do.

The Medication Appropriateness Index (MAI) showed that when the interventions were accepted, there was an important improvement. HSS mean MAI values pre-intervention: 2.52, post-intervention 0.80. In EARs: 5 pre and 1.39 post. In both cases *p* < 0.0001.

**Conclusions:**

Approaching the deployment of activities in a progressive way has made us more efficient and able to confront and solve the problems that have arisen. Even though there has been a very restricted increase in the staff and budget, we are able to implement a DRP detection programme with guaranties of quality.

## Background

The implementation of pharmaceutical care in different levels of health care has been ongoing for several decades. Much work has been completed by pharmacists during this time, even before the first descriptions and terms related to this health process were published by Brodie in the ‘70s and ‘80s [1, 2] and Helper in the ‘90s [3]. In 1993, the American Society of Health-System Pharmacists (ASHP) [4, 5] published several very useful statements for the understanding and implementation of pharmaceutical care in hospitals. However, even though there have been important advances in this area of pharmacy, there are still barriers for its implementation that are more evident in the area of geriatric health care [6–8]. The development of pharmaceutical care has been greater in acute care facilities; this could be due to different causes, for example, the long-term presence of a pharmacy service, more numerous staff and a longer tradition of collaborations between pharmacists and other professionals [9–11].

Grup Mutuam is a group that manages different levels of health care institutions for older people, which include Palliative home care, Long-term and subacute care hospitals (HSS), Nursing homes and Health care teams attending nursing homes (EARs), formed by physicians and nurses.

The implementation of pharmaceutical care has been an important challenge for pharmacy services due to the complexities of the institution, geriatric patients who are polymedicated and have multiple comorbidities and a reduced budget and staff.

Therefore, to establishing the programme of pharmaceutical care in our institutions, we managed in different stages of increasing complexity. That has led us to approach progressively the problems found during the implementation that included, among others, software needs, organization of processes, improvements in some pharmacist’s skills and relationships with other professionals.

**Fig. 1:**
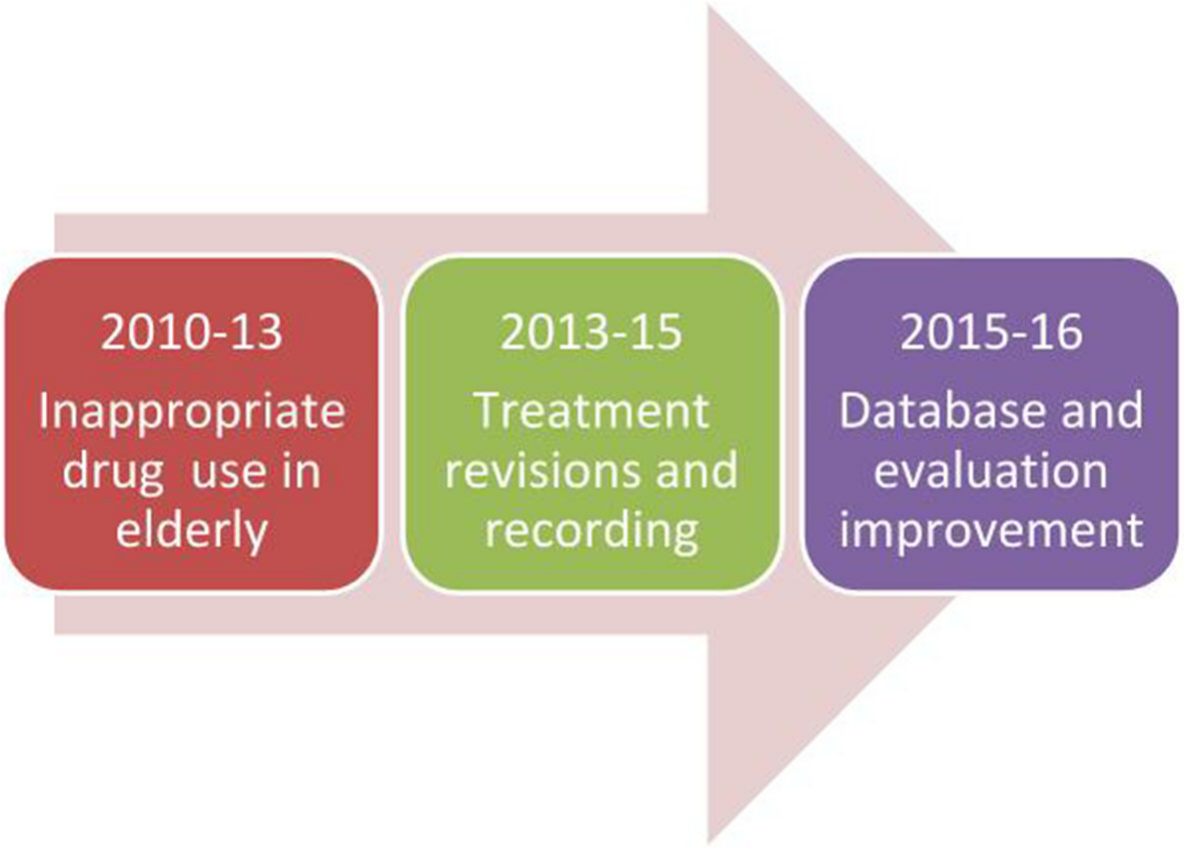
Main steps of pharmaceutical care programme development

In the present revision, we describe the progression of our pharmaceutical care programme initiated on 2010, centred on pharmaceutical interventions, as an experience of how to approach its implementation in a complex geriatric institution (Fig. 1). The development has included an increase in the activities performed by the pharmacists as well as an increase in the number of institutions/patients included in the programme.

The main objectives of the present work were to describe the implementation of a pharmaceutical care program, centered in the revision of pharmacological treatments in pluripathological and polymedicated geriatric patients; and improve collaborations with other professionals, especially physicians, as well as to demonstrate how the detection of inappropriate medication by pharmacists could contribute to reduce their use,

## Methods

The present study is the retrospective-description of the implementation of pharmaceutical care activities in increasing complexity stages. Focused in pharmacist’s interventions to improve the quality of prescriptions, from 2010 till 2017. During this period there were deployed different activities and implemented different tools in order to organize the pharmaceutical activities of our pharmacists, that evolved in sophistication and reliability.

Setting: Different geriatric institutions of Grup Mutuam. That include: Long-term and subacute care hospitals (HSS); Nursing homes; and Health care teams attending nursing homes (EARs), formed by physicians and nurses.

Pharmacist interventions initially were recorded using Microsoft Excel®, and after with the help of a consultant, a business intelligence-based system interconnected with PowerPivot®, was developed and used.

Interventions were classified using the American Society of Health-System Pharmacist (ASHP) classification [12].

For each stage of the program implementation, to assess the impact of pharmacist’s intervention on the quality of drug prescriptions we used the Medication Appropriateness Index [13]. MAI criteria consists of 10 questions, which are graded according to the suitability of the medication (a higher score indicates worse status) and different aspects related to prescription (indication, efficacy, safety and cost).

During all the stages, the pharmacist’s interventions were done according to the recommendations and guidelines of the Spanish Society of Hospital Pharmacy [14] and the (ASHP) [12].

## Results

### Period 2010–13: Inappropriate drug use in elderly

The first activities of pharmaceutical care carried out by Grup Mutuam started in 2010 with a programme directed at reducing the prescription of inadequate medication in elderly people according to the Beers Criteria [15], in two intermediate hospitals (HSS) of the Group, with 165 and 95 beds per hospital.

Development of the program were based in a retrospective evaluation of the quality of the prescription in relation to the Beers Criteria before intervention was conducted in 854 patients who had been admitted into the hospitals during the second half of 2010. During 2011, different actions were completed to inform physicians and nurses about the project. These included the creation of an informative brochure and meetings with physicians and nurses. Intervention stage, March–November 2012 (1332 patients): Prescriptions were reviewed by a clinical pharmacist, and when an inappropriate drug was found, this was communicated to the physician (email or telephone), suggesting a solution. The main results of this study [16] are shown in Tables 1 and 2.

**Table 1:** Main results of the prospective study on the prescription of inappropriate drugs: Inappropriate drugs prescribed before and after the interventions and degree of acceptance of the interventions

	Percentage
Degree of prescription of inappropriate drugs before the intervention	19%
Degree of prescription of inappropriate drugs during the intervention	14.5%
Degree of acceptance of the pharmacists’ recommendations by physicians	45.5%

**Table 2:** Main results of the prospective study on the prescription of inappropriate drugs: Reasons of not acceptance of the interventions

Reasons for non-acceptance	Percentage
Drug prescribed by a specialist	69%
No answer or justification was given	23%
Antibiotic with few alternatives	4%
Dosage adjustment was performed	4%

Patients´ characteristics: ≥ 85 years old, ≥ 9 drugs; 67.5% female

At that time, the low feedback from the physicians it was as expected, as there was little previous experience of working along with them; therefore, this study was an important step in the integration of the pharmacists as a consultant in the health care team of the hospitals.

### Treatment revisions and recording of the interventions: 2013–15

During the work on the detection of inappropriate drugs, we were aware that we needed to broaden the process of prescription validation, directing more efforts toward treatment revisions. At the same time, we were aware that each pharmacist in our staff did this process in different ways, taking into account different issues, and therefore we were not working in a homogenous way. At this point, we also added into the programme two pharmacists who worked in the EARs and began performing revisions of the treatments of patients upon admission to the nursing homes attended by our teams.

All this considered, the next step was to establish a standardized way for performing treatment reviews. We worked on what we considered two basic tools, the elaboration of a therapeutic interchange guideline that was approved by the Pharmacy and Therapeutic Committee and the establishment of an algorithm as a guideline for performing treatment reviews.

**Fig. 2:**
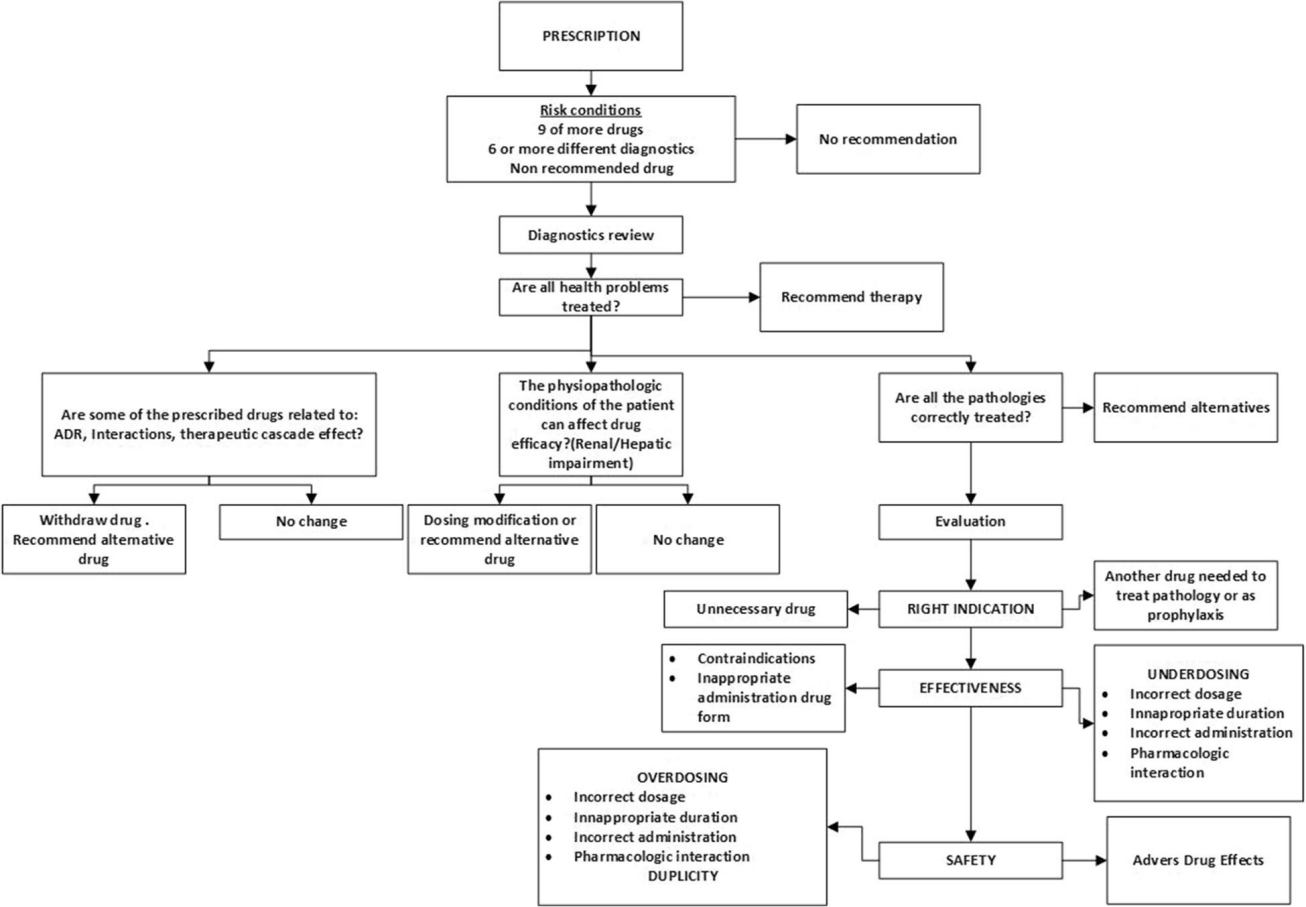
Algorithm for treatment revisions

The algorithm is based on bibliographic information and our day-to-day problems (Fig. 2) [17], and its main objective is to rationalize and unify the review of treatments by pharmacists. It was elaborated by considering and using different resources: Beers Criteria and STOPP-START, establishment of a therapeutic exchange programme, assessment of algorithms of other institutions and review meetings-evaluation of proposals with other professionals. The criteria on which the algorithm is based included the determination of inappropriate medication (medication unnecessary therapy/need additional therapy) applying Beers Criteria [18] and STOPP-START criteria [19]; the effectiveness of the treatment based on the verification of the suitability of the form of administration for geriatric patients, contraindications, verification of the dosage and detection of drug-drug and drug-food interactions; the last determinant was based in patient safety through the adaptation of dose to risk and narrow medication therapeutic range and detection of drug-drug and drug-food interactions.

When a drug-related problem (DRP) is detected, depending on its severity, the pharmacist contacts the physician and/or nurse via telephone or email. The DRP is discussed with the professional, and suggestions are given for solving it.

The interventions/recommendations performed by the pharmacists in the different health care levels managed by Group Mutuam were recorded in a database in Microsoft Excel^•^.

One important but difficult point is to determine the possible benefit in patient outcomes derived from the pharmacists’ interventions. Numerous publications in the literature have tried to approach this by evaluating different items such as the reduction of drug adverse effects or the reduction of length of hospitalization as economic benefits. However, it is still not clear how to evaluate these items or their significance [20, 21].

In our case, as we were in an initial process of establishing the programme, we opted to use the Medication Appropriateness Index (MAI) [13] to evaluate if our interventions had an impact on the prescription quality. Some results of this period are shown in Tables 3, 4 and 5 [22].

**Table 3:** Main results of pharmacists’ interventions (January–December 2014); Institutions included (2 HSS: 268 beds; EARs: 176 nursing homes): Patient characteristics and general data of the interventions, and most prevalent DRP derived from interventions in EARs

Number of pharmacist interventions	1621	
Degree of acceptance	74.5%	
Patient characteristics	Mean age: 86.2 69,5% female	
Mean interventions per patient	1.8	
Most prevalent DRP	Inappropriate drug use in elderly	19.4%
	Condition for which no drug is prescribed	16.4%
	Medication with no indication	15.6%
	Inappropriate dose, form, schedule, route or administration method	12.8%

**Table 4:** Main results of pharmacists’ interventions (January–December 2014) Institutions included (2 HSS: 268 beds; EARs: 176 nursing homes): Patient characteristics and general data of the interventions, and most prevalent DRP derived from interventions in HSS

Number of pharmacist interventions	2251	
Degree of acceptance	81.4%	
Patient characteristics	Mean age: 80.5 60,8% female	
Mean interventions per patient	2.2	
Most prevalent DRP	Inappropriate dose, form, schedule, route or administration method	29.3%
	More cost-effective alternative	19.6%
	More cost-effective alternative	8.4%
	Inappropriate drug use in elderly	8.3%

**Table 5:** Main results of pharmacists’ interventions (January–December 2014) Institutions included (2 HSS: 268 beds; EARs: 176 nursing homes): Main MAI values per patient before and after intervention (for the interventions accepted)

	MAI pre-intervention	MAI post-intervention
EARs	4,83	3,09
HSS	3,68	2,01

In both cases, p < 0,0001 (t-Student applied)

In addition to the improvements obtained, we can also observe that in the HSS, there has been an increase in the acceptance rate of interventions.

### Improvement in the register and data evaluation: 2015–16

Some of the inconveniences found during the implementation of the treatment revisions were the homogenization of the data recorded, even though a unique Excel database had been defined. Moreover, we also faced some difficulties when grouping and classifying the interventions recorded. In addition, the registration of the numerous interventions in Excel was time-consuming, and the possibility of the different pharmacists working in it contemporaneously was complicated or not possible.

Therefore, in this step of the development of our pharmaceutical care program, we focused on the following:

1. Finding a more suitable classification to organize the different types of interventions performed; and
2. Developing a business intelligence-based system to improve data collection and evaluation.

#### Classification of the interventions

After evaluating different classifications in the literature, we opted to use the classification of the American Society of Health-System Pharmacists (ASHP) [12], as that was the one that best fit our needs.

#### Development of a business intelligence-based system

With the help of an informatics consultant, we developed a business intelligence-based system interconnected with Power Pilot® software for recording our data.

The software allows pharmacists to work in the same database at the same time, to access all the drugs and ATC groups, and to be able to update it and to evaluate in an easy way, as well as to have results in real time.

In Tables 6, 7, 8, and 9, we show several results.

**Table 6:** Main results corresponding to the period January–December 2016: Number and characteristics of the patients in which DRP were found

Patient characteristics	
n	3630
Sex	64.8% Female
Mean age	83.2 years old (±9.7) (range 108–34)

**Table 7:** Main results corresponding to the period January–December 2016: Number of interventions and physicians acceptance

Interventions	
Number	5903
Mean interventions/patient	1.62 (range 18–1)
Mean number of medicines/patient	8.4 (SD: ±3.3)
Acceptance of the interventions by physicians	68.6%

**Table 8:** Mean MAI values pre- and post-intervention (for accepted interventions)

	MAI pre-intervention	MAI post-intervention
EARs	5 (±6.1)	1,39 (±3.73)
HSS	2,52 (±3.2)	0,80 (±2.20)

In both cases, p < 0,0001

**Table 9:** Percentage of the main drug-related problems (DRP) detected that generated pharmacist interventions. (IR: Renal Insufficiency)

Drug related problems detected	HSS	EAR
PROBLEMS ARE ARISING FROM THE FINANCIAL IMPACT OF THERAPY	14,2	6,9
CONDITION FOR WHICH NO DRUG IS PRESCRIBED	11,9	10,2
THERAPEUTIC DUPLICATION	10,2	8,8
MEDICATION PRESCRIBED INAPPROPRIATELY FOR A PARTICULAR CONDITION	9,8	18,6
LENGTH	9,0	21,3
SEQUENCIAL THERAPY	7,2	0
SCHEDULE	6,5	2,5
INAPPROPRIATE DOSE	5,8	3,6
LACK OF UNDERSTANDING OF THE MEDICATION	5,6	1,68
DOSAGE FORM	3,5	0,14
ROUTE OF ADMINISTRATION	3,1	0,1
MEDICATION WITH NO INDICATION	3,0	15,5
METHOD OF ADMINISTRATION	2,7	0,1
DRUG-NUTRIENT THAT ARE CLINICALLY SIGNIFICANT	2,3	0
INAPPROPRIATE DOSE IR	1,9	2,7
ACTUAL AND POTENCTIAL DRUG-DRUG THAT ARE CLINICALLY SIGNIFICANT	1,4	0,5
ACTUAL AND POTENTIAL ADVERSE DRUG EVENTS	0,9	2,5
FAILURE TO RECEIVE THE FULL BENEFIT OF PRESCRIBED THERAPY	0,4	2,86
DRUG-DISEASE THAT ARE CLINICALLY SIGNIFICANT	0,1	1,28

Institutions included in the current program:

- HSS: 3 institutions with a total of 291 beds.
- Nursing homes (EARs): 237 nursing homes (13,105 residents).

## Discussion

The incorporation of the pharmacist in the clinical team in geriatric institutions is not as widespread as in acute care. This is manifested with our initial results of the acceptance of our interventions in the first period of developing our programme, and even though there are advances we have made in these years as include the clinical pharmacist in geriatrics team works and improve the results regarding prescriptions, there is still much work to do. The degree of acceptance during 2016 was lower vs that of 2014, but we have to take into account that the number of interventions has increased due to the incorporation of the clinical pharmacists in the new EARs teams and increased HSS, where the pharmacists had no previous relationships with the physicians.

As expected, we found more difficulties in nursing homes than in hospitals because the existing interactions between pharmacists and physicians were lower.

As it has been seen in many other institutions like ours, DRP are numerous, and the quality of the prescriptions of the patients at admission is somewhat poor, including medication errors. During these years, we have seen that medication revision is important, not only at admission and at discharge (reconciliation) but also during the stay, although all of this contributes to increasing the workload of the pharmacists. Therefore, to optimize and increase the effectiveness of the revisions, it is essential to have a structured method of working, including a normalized and automated recording of the interventions. To have the results of the activities performed by the pharmacists is fundamental in showing managers and other professionals the value of our work.

Assessing the MAI values when arriving at our institutions through the years, we have detected that the quality of the prescription has slightly improved in HSS. This could be due the fact that the majority of patients in HSS are admitted from acute-care hospitals where pharmacy services are doing an important job in medication reconciliation. In addition to this, in the MAI values across the years, we can see that the results of our work are more effective because during 2016, we obtained a higher improvement in the MAI values.

Considering all the evolution of our work we think that main achievements have been to be able to agree between all the pharmacists in a unique algorithm, that unifies the way of making treatment revisions in our patients. Other positive achievements have been to agree in a classification of the interventions and the development of a software for recording and exploiting our data. At the beginning, we employed a lot of time developing the Excel database for recording our data, that could have been avoided if we would have asked the technical support that lead later in the development of the software.

For us, after having implemented an effective program for medication review and pharmacist interventions, our main objectives are to continue increasing the level of answers and acceptance by physicians and to be able to determine the impact of our interventions in patient outcomes, as well as to focus more on the most prevalent DRP. This requires a combination of improving relationships, showing the benefits derived from the acceptance of our recommendations, and finding more effective communication methods to help physicians answer us with a minimum disruption of their work.

The number of patients attended in our institutions continues to grow as well as their complexity, and the workload of revisions is increasing; therefore, we need tools to detect which patients to focus on. Thus, we are now also directing our project in two different ways. First, we are trying different validated tools [23] for detecting patients at higher risk, and second, we are increasing our relationships with the pharmacists in acute-care hospitals and primary care, where the majority of our patients are coming from, to work in a territorially integrated way.

## Conclusions

Choosing a stepwise process is very useful in the initiation of a pharmaceutical care programme in geriatric institutions with low integration of pharmacy services.

A standardization of how to perform the reviews is necessary when different pharmacists in different institutions have to work with the same aim.

A record with a comprehensive classification of interventions is fundamental to reduce workload and maintain data about pharmacists’ work.

Technological tools are paramount for keeping the programme sustainable and being able to confront an increase in patients and their complexity.

## Abbreviations

ASHP: American Society of Health-System Pharmacists
DRP: Drug Related Problems
EARs: Health care teams attending nursing homes
HSS: Long-term and subacute care hospitals
MAI: Medication Appropriateness Index
